# Highly elevated serum levels of CA 19-9 in choledocholithiasis: a case report

**DOI:** 10.4076/1757-1626-2-6662

**Published:** 2009-07-30

**Authors:** Georgios Marcouizos, Eleftheria Ignatiadou, Georgios E Papanikolaou, Dimosthenis Ziogas, Michail Fatouros

**Affiliations:** Department of Surgery, University Hospital of Ioannina, School of MedicineIoanninaGreece

## Abstract

We present a case of a 79-year-old woman admitted to our hospital with pain in the right upper abdominal quadrate radiated to the back, jaundice, fever and chills. The laboratory tests showed serum carbohydrate antigen 19-9 levels of 99.070 U/ml (normal values: 0-37 U/ml). The rest of the biochemistry showed alkaline phosphatase of 550 IU/l, direct bilirubin: 17.5 mg/dl, total bilirubin: 28.4 mg/dl. Abdominal sonography demonstrated dilated common bile duct. Two weeks postoperatively, the carbohydrate antigen 19-9 fell to 970 U/ml and returned within normal range (31 U/ml) two months later. Furthermore, the magnetic resonance cholangiopancreatography performed postoperatively demonstrated normal configuration of the biliary tree and the common bile duct.

## Introduction

Carbohydrate Antigen (CA) 19-9, discovered by Koprowski et al [1] is a gastrointestinal cancer-related antigen measured by a monoclonal antibody, which shows a high positivity in pancreato-biliary malignancies. However, high levels of serum CA 19-9 is occasionally found in benign disease of the liver, pancreas and biliary tract, especially in cases with gall stone disease in which the high rate of its elevation has been reported in acute stage, but the value is usually below 5.000 U/ml [2,3]. We report a case of common bile duct stone with acute cholangitis presenting with extraordinarily high serum CA 19-9 levels, which returned to the normal after surgical removal of the stone.

## Case presentation

**Figure 1.**
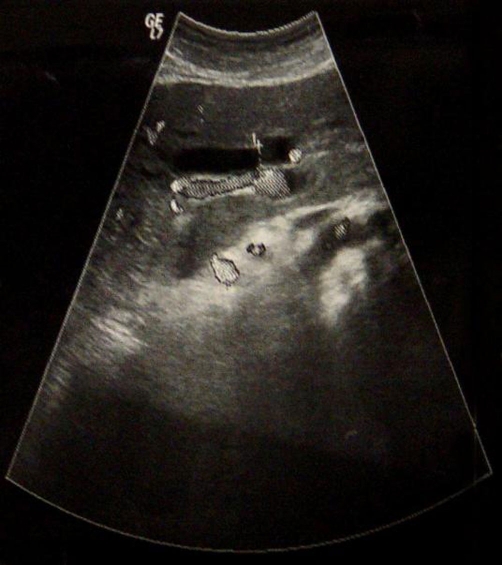
Abdominal sonography showed dilatation of the common bile duct.

A 79-year-old woman Caucasian woman was admitted with the chief complaints of epigastralgia with radiation to the back and jaundice for about ten days. Fever and chills were noted for seven days. The laboratory data were as follow: white blood cell count of 12.020/μl (normal values: 3900-10600/μl), alanine aminotransferase: 350 IU/l (normal range: 10-35 IU/l), alkaline phosphatase: 550 IU/l (normal range: 30-125 IU/l), direct bilirubin: 17.5 mg/dl (normal values: 0.01-0.2 mg/dl), total bilirubin: 28.4 mg/dl (normal values: 0.1-1 mg/dl), CA 19-9: 99.070 U/l (normal value: 0-37 U/l). Abdominal sonography showed dilatation of the common bile duct (maximum diameter: 1.3 cm) (Figure 1).

**Figure 2A and B.**
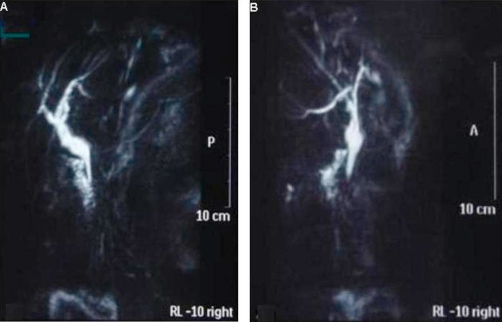
The MRCP performed two months postoperatively demonstrated normal configuration of the intrahepatic biliary tract and the common bile duct.

During the operation, cholecystectomy and choledochotomy were performed. Upon opening the dilated common bile duct choledochoscopy was done and a stone was noted and removed at the distal common bile duct measured about 1.2 cm in diameter. The common bile duct was closed with T-tube. Two weeks after the operation, the CA 19-9 fell to 970 U/ml and returned to the normal range (31 U/ml) two months later. Postoperatively, the magnetic resonance cholangiopancreatography (MRCP) showed normal configuration of the biliary tree and the common bile duct (Figure 2A,B).

## Discussion

CA 19-9, carbohydrate antigen that was defined by monoclonal antibody to the cultured cell from human colonic cancer, was termed by Korprowski in 1979 [1]. Initially was considered to be a tumor marker associated with colon cancer, but later became a useful tumor marker for pancreatobiliary malignancies.

CA 19-9 is produced in normal human pancreatic and biliary ductal cells. Since the exact pathway between tissue and blood is still not well known, the real mechanism of the elevated serum CA 19-9 remains unsettled [4]. A CA 19-9 value of more than 1.000 U/ml usually indicates a gastrointestinal cancer and has been reported to have specificity greater than 99% for pancreatic cancer, nevertheless, false-positive results can be found to benign disease such as pancreatitis or liver cirrhosis [5]. Also, is well known that elevated CA 19-9 in the sera may occur in various benign diseases such as gall bladder stone disease, chronic pancreatitis, hepatitis, liver cirrhosis, renal failure, duodenal ulcer, gastric polyps, colonic polyps and renal cyst.

The percentage of patients with a serum CA 19-9 level more than 10.000 U/ml is reported to be 6-31% in pancreatic cancer and 0-30% in biliary tract cancer [6]. There are few reports however, of gall stone patients with such a high serum levels of CA 19-9. There have been only two cases of common bile duct stone with a serum CA 19-9 level greater than 10.000 U/ml in the literature [2,7].

Surprisingly, extremely elevated serum CA 19-9 in patients with acute cholangitis like the reported case may occur. Murohisa et al [8] presented a case of bile duct stone with cholangitis and high serum CA 19-9 level (60.000 U/ml), which returned to the normal 6 weeks later. In our reported case, the serum CA 19-9 value returned to the normal two months later.

Production and secretion of CA 19-9 from malignant cells are considered to be responsible for the high serum CA 19-9 level in malignancies. The real reason for the CA 19-9 elevation in acute cholangitis is not clear. Several mechanisms have been postulated as: 1) leakage of condensed CA 19-9 due to biliary tract obstruction from the bile into blood circulation [9], 2) CA 19-9 production by irritated bile duct cells exposed to increased biliary pressure may be enhanced, 3) enhanced production of CA 19-9 in the bile duct epithelium and the mucosa of gall bladder induced by the inflammatory process, 4) the inflammatory cytokines produced in sepsis due to cholangitis probably have some contribution.

Though the presence of a gastrointestinal cancer may enhance the production of CA 19-9, extreme elevation of CA 19-9 could occur in benign biliary obstruction and inflammation as in our reported case. The precise mechanism for this abnormal high levels remains to be clarified.

## Abbreviations

CA: Carbohydrate Antigen
MRCP: Magnetic Resonance Cholangiopancreatography

## References

[bib-001] Koprowski H, Steplewski Z, Mitchell K, Herlyn M, Herlyn D, Fuhrer P (1979). Colorectal carcinoma antigens detected by hybridoma antibodies. Somatic Cell Genet.

[bib-002] Barone D, Onetto M, Conio M, Paganuzzi M, Saccomanno S, Aste H, Pugliese V (1988). CA 19-9 assay in patients with extrahepatic cholestatic jaundice. Int J Biol Markers.

[bib-003] Steinberg W (1990). The clinical utility of the CA 19-9 tumor-associated antigen. Am J Gastroenterol.

[bib-004] Arends JW, Verstynen C, Bosman FT, Hilgers J, Steplewski Z (1983). Distribution of monoclonal antibody-defined monosialoganglioside in normal and cancerous human tissues: an immunoperoxidase study. Hybridoma.

[bib-005] Craxi A, Patti C, Aragona E (1985). Serum CA 19-9 levels in patients with hepatocellular carcinoma or cirrhosis. Ital J Gastroenterol.

[bib-006] Oguchi H, Homma T, Nagata A, Kawa S, Hirabayashi H, Tamura Y, Monno S, Shirai T, Shimakura K, Koike Y, Matsuda Y, Furuta S, Shiga T, Hayashi S, Miyazawa K (1984). Monoclonal antibody-defined tumor marker CA 19-9: evaluation of its clinical usefulness, using the radioimmunometric assay for CA 19-9 (in Japanese). Nippon Shokakibyo Gakkai Zasshi.

[bib-007] Sheen-Chen SM, Sun CK, Liu YW, Eng HL, Ko SF, Kuo CH (2007). Extremely elevated CA 19-9 in acute cholangitis. Dig Dis Sci.

[bib-008] Murohisa T, Sugaya H, Tetsuka I, Suzuki T, Harada T (1992). A case of common bile duct stone with cholangitis presenting an extraordinarily high serum CA 19-9 value. Intern Med.

[bib-009] Lim BC, Park ET, Yoo KS, Seo DW, Lee SK, Min YI (1999). A new strategy for the application of CA 19-9 in the differentiation of pancreaticobiliary cancer: analysis using a receiver operating characteristic curve. Am J Gastroenterol.

